# Tomographic evaluation of dentoskeletal effects of rapid maxillary expansion using Haas and Hyrax palatal expanders in children: A randomized clinical trial

**DOI:** 10.4317/jced.57277

**Published:** 2020-10-01

**Authors:** Marília-Carolina Araújo, Jéssica-Rico Bocato, Paula-Vanessa-Pedron Oltramari, Marcio-Rodrigues de Almeida, Ana-Cláudia-de Castro-Ferreira Conti, Thais-Maria-Freire Fernandes

**Affiliations:** 1 D.D.S., M.Sc. UNOPAR - University of North Paraná, Brazil; 2 D.D.S., M.Sc. PhD. Assistant Professor, Department of Orthodontics, UNOPAR - University of North Paraná, Brazil

## Abstract

**Background:**

Rapid maxillary expansion (RME) is a usual procedure for correcting the transversal maxillary deficiency. Among the most used appliances are the Haas type (tooth-tissue-borne) and Hyrax (tooth-borne) whose main difference is the design. This study aimed to evaluate the dentoskeletal effects of RME using two different expanders in children.

**Material and Methods:**

The sample was composed of 42 children of both gender presenting unilateral or bilateral posterior crossbite with mean age 9.49 (SD± 1.35). Patients were randomized into two groups according to the type of expander: Hyrax (n= 21, 9 boys and 12 girls) and Haas (n= 21, 11 boys and 10 girls). Multiplanar coronal and axial slices obtained from cone-beam computed tomography images (i-Cat, Hartsfield, PA, USA) were used at pretreatment (T1) and after 6 months when the expander was removed (T2). Measurements were performed on Dolphin Imaging Systems 11.7 software (Chatsworth, California, USA). The following variables were evaluated: inclinations of the posterior teeth, transverse skeletal widths, length of maxillary dental arch, buccal bone thickness and level of buccal alveolar crest. Statistical analysis performed using chi-squared test to compare the sex ratios between groups and independent t test with the Bonferroni correction for multiple tests.

**Results:**

RME increased all maxillary transverse dimensions, regardless of the type of expanders used. Subjects in the Hyrax group experienced significantly increase in the lingual bone thickness (0.94 mm) compare to Haas group (0.21 mm).

**Conclusions:**

The Hyrax-type expander produced greater increase in the lingual bone thickness than did the Haas-type expander, but this effect might not be clinically significant. Both appliances presented similar transversal gain and tended to produce similar orthopedic and orthodontic effects.

** Key words:**Cone-beam computed tomography, palatal expansion technique, palate.

## Introduction

Rapid maxillary expansion (RME) is a usual procedure for correcting the transversal deficiency in upper arch, aiming to increase the perimeter of the maxillary with rupture of the midpalatal suture by using expanders (1-3). This process occurs due to the position of the expander screw parallel to the suture. The activation is quick and aims to accumulate force to break the resistance imposed by the suture (4).

An early treatment, in the mixed dentition stage, is suggested due to greater bone elasticity, less resistance to expansion and consequently less painful symptomatology (5). Among the most used appliances are the Haas type (tooth-tissue-borne) and Hyrax (tooth-borne) whose main difference is the presence or absence of acrylic pad close to palate (2,6).

The advantages of the tooth-borne expanders are the easy hygienic, the greater comfort and the prevention of injuries in the soft-tissue (7), whereas with tooth-tissue-borne expander, there is the possibility of greater expansion at the base of the maxilla (8). In addition, the absence of acrylic pad with exclusive dental support may allow recurrence of orthopedic effects. Although a cephalometric (9), occlusal (10) and frontal radiographs (11), and conventional computed tomography (6) investigation have not demonstrated differences between Haas type and Hyrax expanders, there is no consensus in the literature regarding the differences in the 6 months RME changes produced (12).

In addition, two systematic reviews (13,14) have shown that the available studies show poor quality and insufficient evidence to determine a difference between any type of cross-bite treatment expanders (13). Therefore, this randomized clinical trial was designed to evaluate the differences in the dentoskeletal effects of rapid maxillary expansion using cone-beam computed tomography (CBCT) between two different appliances (Hyrax and Haas type) after a stability period of 6 months.

The purpose of this study was to evaluate the dentoskeletal effects of the Hyrax and Haas type expander after stability period of treatment. The null hypothesis was that there are no differences between the expansion appliances in children.

## Material and Methods

**Figure 1:**
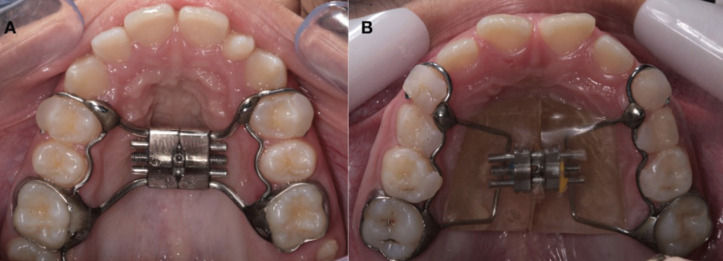
Occlusal photographs showing the expanders: A, Hyrax; B, Haas type.

This study was previously approved by the Institutional Review Board of the University of Northern Paraná (UNOPAR) / Plataforma Brasil (2,008,872) and registered on the Brazilian clinical trials register site (U1111-1185-7694). Parents signed the informed consent form before the intervention. The participants of each group were randomly into 2 groups (1:1 allocation ratio) and treated with two types of expanders, Hyrax and Haas (Fig. 1). The patients were treated by 2 Orthodontists residents and supervised by a faculty member. No changes in methods occurred after trial began.

A sample of patients presenting unilateral or bilateral crossbite was recruited through the evaluation of scholar children and patients were asked to attend, with their parents, the Dental Clinic of the University of Northern Paraná, Londrina, Brazil for a new evaluation in a clinical setting. Inclusion criteria included children presenting unilateral or bilateral posterior crossbite. Individuals who had craniofacial anomalies, compliance problems, periodontal disease, agenesis and supernumeraries teeth, anterior crossbite or open bite, permanent tooth losses, extensive cavities and previous orthodontic treatment history were excluded.

In both groups, screws were activated with a complete turn a day until achieving an overcorrection at the molar region, with the palatal cusp tip of the maxillary posterior teeth contacting the buccal cusp tip of the mandibular posterior teeth (approximately 7 mm). Parents were daily notified by activation time through the WhatsApp Messaging application so that there was better treatment outcome, according to Leone el al. (15), text messages are a positive influence on patient cooperation. After the active phase of treatment, the appliances remained as retainer for a period of 6 months.

The CBCT images were acquired at pretreatment (T1) and at after stability period of expansion (T2). Images were captured on the i-CAT (Imaging Sciences International, Hatfield, Pa), with exposure parameters of 120 kVp, 40 seconds, field of view of 8 cm, and voxel size of 0.3 mm. The position of the patient’s head was standardized so that the Frankfort plane was parallel and the midsagittal plane was perpendicular to the ground (Natural head position). The DICOM files were measured by 1 single examiner (M.C.A.) using the Dolphin Imaging Systems 11.7 program (Chatsworth, California, USA) blindly.

**Figure 2:**
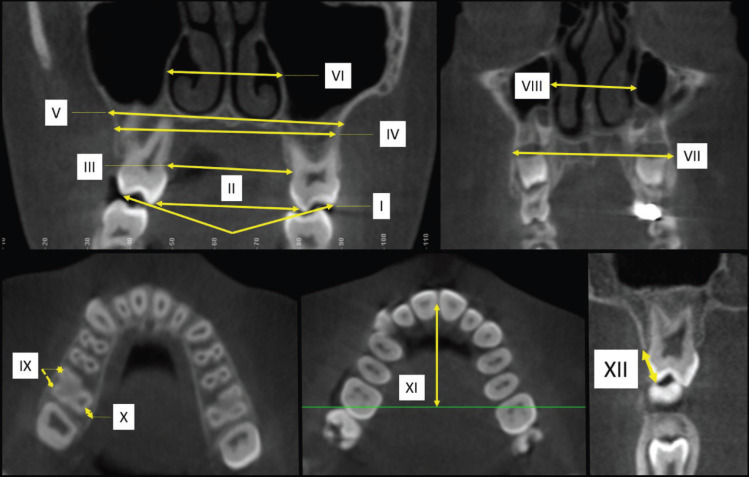
CBCT images showing the measurements of the different variables: A, Axial slices (posterior region): I - Tooth inclination, II - arch width (lingual) III - alveolar crest width (lingual), IV - maxillary width (external cortical), V – maxillary width (in the floor or the nasal cavity), VI - nasal cavity; B, Axial slices (anterior region): VII - Maxillary width, VIII - nasal cavity width; C, Coronal Slices (posterior region): IX - Thickness of the buccal (mesial and distal), X - thickness of the lingual bone plate; D, Coronal Slices (posterior region): XI - Arch perimeter; E, Parassagital images (posterior region): XII - level crest bone.

CBCT scans were reoriented as perpendicular to the midpalatal suture (axial slice), parallel to the palatal plane (ANS-PNS, sagittal slice), and tangent to the nasal floor at its most inferior level (coronal slice). Coronal slices were used to measure in posterior region: Arch perimeter, thickness of the buccal (mesial and distal) and lingual bone plate. Axial slices were used to measure in posterior region: Maxillary width (external cortical and in the floor or the nasal cavity), nasal cavity width, alveolar crest width (lingual), arch width (lingual) and tooth inclination. Anterior region: Maxillary and nasal cavity width. In addition, parassagital images were obtained by registering on the level crest bone in posterior region (Fig. 2).

Before measurements the image was standardized, respecting the Frankfort plane and the orbital plane, perpendicular to the midsagittal plane. For the axial section, the position of the nasal septum was adopted in its most superior portion, perpendicular to the horizontal plane.

The primary outcome of the study was the correction of crossbite with the palatal cusp of the maxillary first molar touching the buccal cusp tips of the mandibular first molar.

The transverse dimensions and posterior teeth inclinations obtained the measurements on tomographic images. Transverse dimensions of the maxilla were measured in 2 coronal images perpendicular to the midsagittal plane, the first one passing through the center of the palatal root of the maxillary right permanent first molar (posterior region) and the second, displaced 15 mm anterorly (anterior region).

Figure 2 shows some linear variables obtained in the coronal images. A buccolingual inclination of the maxillary posterior teeth was measured only in the posterior region. Whereas the level of the buccal bone crest of the supporting teeth was measured by means of parasagittal images.

-Sample Size Calculation

Calculation of sample size was based on the ability to detect a difference in maxillary width of 1.1 mm (SD, 1.10), measured between the external cortical to the level of the deepest region of the palate (16), with an alpha of 5% and a test power of 80%.

Sixteen individuals would be required in each group. Twenty-one individuals in the hyrax group and 21 in the Haas group were recruited to ensure the power in case of any dropouts.

The patients were randomly assigned to one of the two treatment groups via a block randomization procedure with a block size of four, using a computer-generated (Microsoft Corporation - Redmond, USA) (17) list of random numbers. The allocation sequence was concealed from orthodontists and patient’s parents. When a patient was deemed as eligible for enrollment, the patient was assigned to a treatment group using opaque and sealed envelopes containing the allocation number.

There was allocation concealment and blinding of outcomes assessment, however no blinding of participants or operators, due to the presence of the appliances.

-Statistical Analysis

According to the Shapiro-Wilk test, the data had normal distribution (*P* > .05). The data were described by mean and standard deviation parameters. To verify the reliability of the measurements, 30% of the CBCT scans were randomly reexamined in 4-week intervals to calculate the error of the study using the Intraclass Coefficient Correlation (ICC) and Bland-Altman agreement. Chi-squared and independent t test were respectively used to compare sex ratio and initial age between groups (*P*<0.05). Intergroup comparisons were performed using independent t test with the Bonferroni correction for multiple tests (t test on a set of 13 CBCT measurements) and for intragroup comparisons dependent t test was used.

The statistical analysis was performed in Statistica 7.0 software (StatSoft Inc., Tulsa, OK, USA). Associated 95% confidence interval (CI) was adopted.

## Results

**Figure 3:**
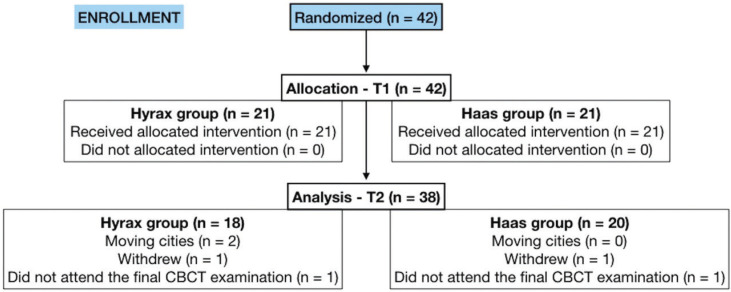
CONSORT diagram showing patient flow during the Trial.

Forty-two children were randomly assigned to two experimental groups: 21 children in the Hyrax group, 9 boys and 12 girls with initial age of 9.67 (SD, 1.64) years old and 21 children in the Haas group, 11 boys and 10 girls with initial age of 9.33 years old (SD, 1.04). With regards to dropouts of the sample, two individuals moved to another city, 1 patient withdrawn from treatment, and 1 patient did not attend the final CBCT scan (Fig. 3).

Participants from both groups were similar in age, sex (Table 1) and dentoskeletal measurements in T1.

**Table 1:**
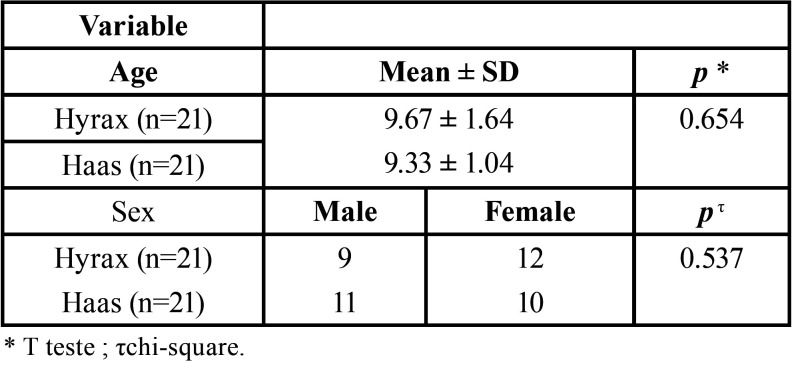
Intergroup comparisions for age and sex ratio (t and chi-square test).

Intra-examiner agreement for the CBCT analysis was excellent, with ICCs ranging from 0.91 to 0.98. The Bland-Altman analysis yielded analogous results, with low bias for all variables and narrow confidence intervals, indicating good replicability of the measurements.

When comparing the changes between the groups (T2-T1) (Table 2), 1 variable presented statistically significant differences (*P* < .01). There was a statistically significant increase in lingual bone thickness in the Hyrax group (0.94mm) compared to the Haas group (0.21mm). There were no significant intergroup differences in the maxillary transversal measurements. Increased nasal cavity width in the posterior region (Hyrax = 2.12 mm; Haas = 1.84 mm) represented 37% of the total arch width expansion (5.60 mm) in the Hyrax group and 33% in the Haas group of the total expansion (5.49 mm).

**Table 2:**
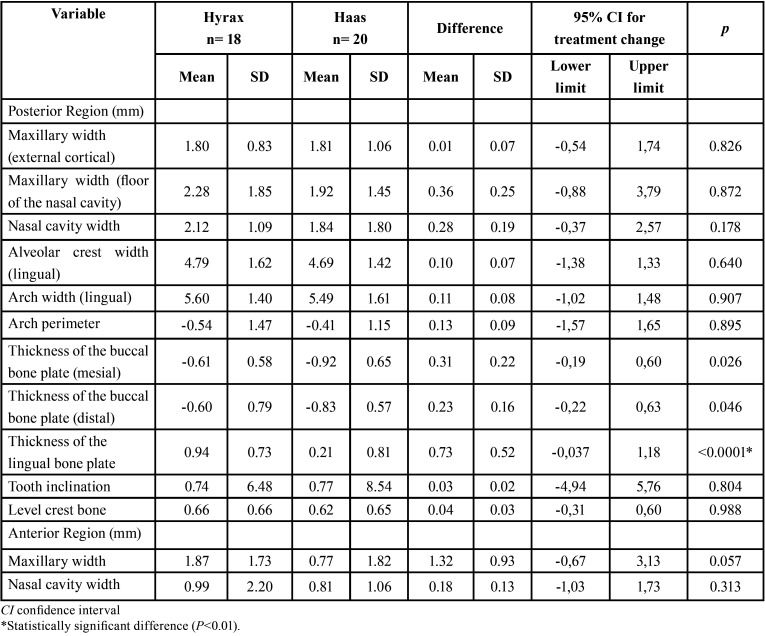
Intergroup comparisons of the expansion changes (T2-T1) (independet t test).

After expansion, there was a statistically significant increase in the Hyrax group in the variables that measured maxillary width and lingual bone thickness and a decrease in vestibular bone thickness (Table 3). Whereas in the Haas group there was no increase in the nasal cavity and maxillary width of the anterior region. The thickness of the buccal bone plate decreased after the expansion (*P*<0.05), but there was no difference in the lingual thickness (Table 4). In both groups there was no statistically significant difference for arch length and tooth inclination ( Table 3, Table 4).

**Table 3:**
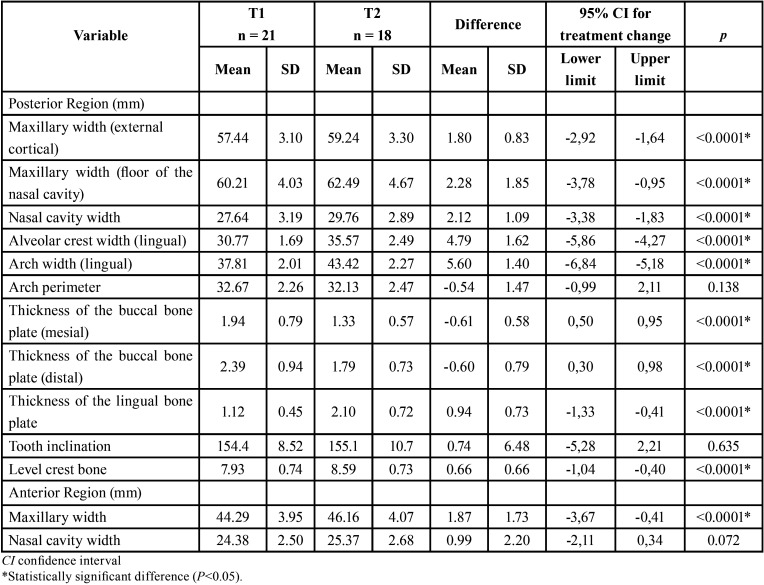
Dentoskeletal effects of rapid maxillary expansion after 6 months for the Hyrax group (intragroup comparison) (t test).

**Table 4:**
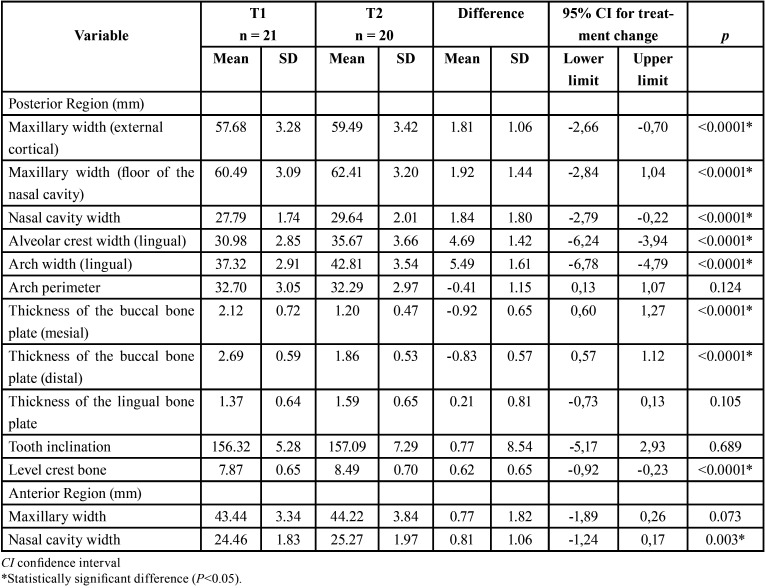
Dentoskeletal effects of rapid maxillary expansion after 6 months for the Haas group (intragroup comparison) (t test).

## Discussion

Main findings in the Context of the Existing Evidence, Interpretation

This study assessed the effects of Hyrax and Haas type expanders on growing and posterior crossbite individuals to verify the dentoskeletal effects of RME in the transversal dimensions by means of the CBCT, a high resolution examination that presents minimal distortion and with lower doses of radiation than conventional tomography (9,11).

Previous studies that used CT to assess the effects of RME have mostly had a small sample size (6,12,18,19). The current study had an adequate sample size (21 patients in the Hyrax group and 21 in the Haas group) and presents important characteristics such as: 1) it was a prospective study; 2) the patients were randomized into the groups; 3) the methodology was standardized in terms of appliance fabrication, and rate and amount of expansion; and 4) high-resolution CBCT was used.

It is usual to report in the literature the movement for buccal anchoring teeth in the rapid maxillary expansion (4,18,20), which is normalized after removal of the expander and corrective orthodontic treatment. Orthodontic and orthopedic forces have been shown to cause histological changes, such as the activation of clastic cells towards the periodontal ligament and hyalinization on the pressure side, and that the lateral inclination of the anchoring teeth may cause bone resorption at the dentoalveolar level (19,20).

In the current study, in the thickness of the lingual bone plate there was a greater increase in the Hyrax group (0.94 mm) in relation to Haas type (0.21 mm). These results were similar to those of Garib *et al.* (6) suggest that the pressure exerted by the acrylic of the Haas expander can stimulate bone resorption in the palatal region of the alveolar process (18,20). However, CBCT with voxel size of 0.3 and 0.4 voxels are ideal for general treatment planning, it should be used with caution if the goal is to assess small variations in bone thickness. (21) Alveolar changes after rapid maxillary expansion were evaluated in two different voxel protocols (0.25 mm and 0.4 mm) and excellent accuracy has been found. Although, when the alveolar bone thickness is close to or smaller than the voxel size (0.4 mm), the measurements were underestimated by 0.9 to 1.2 mm. With these inaccuracies, using 0.4 mm image resolution, the alveolar losses associated with rapid maxillary expansion may be overestimated because the bone is indistinguishable from the periodontal ligament (22). Therefore, our results must be used with caution because of the voxel size used (0.3 mm).

The effect of the expansion decrease in the upward direction, as described in the literature, which means that the maxillary width in the molar region (Hyrax = 5.60 mm and Haas = 5.49 mm) presented a higher gain than in that of the width of the nasal cavity (Hyrax = 2.12 mm and Haas = 1.84 mm).

**Figure 4:**
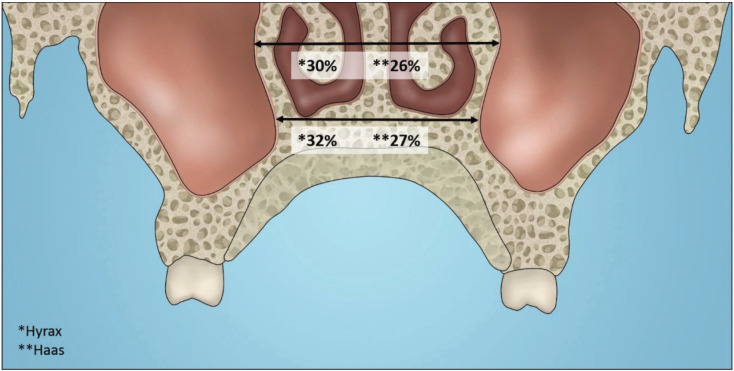
The orthopedic effects: in the width of the nasal cavity and on the floor of the nasal cavity.

The orthodontic changes showed similar gains in alveolar crest width (68%), arch width (80%) and in the deepest region of the palate, the external cortical (25%) of the Hyrax group in relation to the Haas group (67%; 78% and 25%) when taking into account the total expansion (7 mm). The orthopedic effects were slightly higher in the Hyrax expander. In the deepest region of the palate, there was an increase of 32% on the floor of the nasal cavity and 30% in the width of the nasal cavity in the posterior region. All these measures took into account the total expansion (7 mm). In the Haas group, the increase was lower on the floor of the nasal cavity (27%) and in the width of the nasal cavity (26%) (Fig. 4). Weissheimer *et al.* (12) also found greater gains in the Hyrax-type expander compared to Haas, but in a study comparing immediate dentoskeletal effects for the same types of expanders. The fact that the Hyrax expander has produced greater transversal gains can be explained by differences in the design of the expanders. In Hyrax the support connecting mechanism for the bands of the anchoring teeth is with a rigid structure of stainless steel, unlike the Haas type expander, where the acrylic pad is responsible for connecting the stainless steel structure. According to Braun *et al.* (23) on the biomechanics of RME, the expanders with the acrylic pad support are much less rigid than those constructed exclusively of stainless steel wire, as in the case of the Hyrax type expander (23).

This study aimed to evaluate not only the transverse changes of the maxilla, but also skeletal alterations, mainly because the patients were in the growth stage. Our data showed increases in nasal cavity width in the posterior region, and in the Hyrax group the increase was of 2.12 mm representing 30% of the total activation value (7 mm) and in the Haas group of 1,84 mm representing 26%. Garib *et al.* (6) and Christie *et al*. (24) also reported cross-sectional increase at the level of the nasal floor corresponding to one third of the amount of expansion. This finding may support the theory that maxillary expansion increases airflow and improves nasal breathing (20). However, further research is needed to assess how nasal airway volume is modified by RME.

-Limitation

One limitation of this study was the lack of an untreated control group, a problem that occurs in similar studies (6,9,12,18). Because of ethical issues we could not expose patients to unnecessary CBCT radiation and besides that, we would keep them without treatment for 6 months despite their need for immediate intervention. In addition, Moorrees and Reed (25) showed that the increase in intermolar distance is 3 to 4 mm from 6 to 17 years of age (0.36/year). So, the transversal changes from growth would be very small in 6 months, which justifies the absence of a control group.

-Generalizability

The generalization of these results may be limited to patients requiring expansion because the effects may differ according to age and type of constriction (unilateral or bilateral, or only atresia). In addition, these results should not be generalized to different types of expanders or to the same expanders used with different activation protocols (7,14,32).

It should also be mentioned that the activation protocol of this study was carried out in the home environment by those responsible for the children, and therefore, we were dependent on their cooperation.

-Interpretation

Thus, it is up to the professional to consider the cost-benefit ratio to indicate which of the expanders should be used to achieve the best results. In this regard, tooth-tissue-borne or tooth-borne appliances may present specific particularities. Both presented a transversal gain, but the characteristics of the bone plate and the presence of mouth breathing should be taken into account during the choice of treatment. Based on our findings, further research is needed to assess the long-term results and stability, as well as the analysis of the side effects of interventions.

## Conclusions

Based on this clinical trial with CBCT to assess the effects of RME on the transverse plane with 2 kinds of palatal expanders after stability period, the null hypothesis was accepted, the following conclusions can be drawn:

The Hyrax expander produced greater increase in in the thickness of the lingual bone plate (0.94 mm) in relation to Haas type (0.21 mm). However, our results must be used with caution because of the voxel size used (0.3 mm).

The Hyrax expander promoted gain in maxillary width (32%) and nasal cavity (30%) similar to Haas type expander (27% and 26%, respectively).

The orthodontic changes showed similar gains in alveolar crest width (68%), arch width (80%) and in the deepest region of the palate, (25%) of the Hyrax expander in relation to the Haas type (67%; 78% and 25%) when taking into account the total expansion (7mm).
